# Revisiting the Diego Blood Group System in Amerindians: Evidence for Gene-Culture Comigration

**DOI:** 10.1371/journal.pone.0132211

**Published:** 2015-07-06

**Authors:** Christophe Bégat, Pascal Bailly, Jacques Chiaroni, Stéphane Mazières

**Affiliations:** 1 Aix Marseille Université, CNRS, EFS, ADES UMR 7268, 13916 Marseille, France; 2 Etablissement Français du Sang Alpes Méditerranée, 13392 Marseille cedex 5, France; University of Florence, ITALY

## Abstract

Six decades ago the DI*A allele of the Diego blood group system was instrumental in proving Native American populations originated from Siberia. Since then, it has received scant attention. The present study was undertaken to reappraise distribution of the DI*A allele in 144 Native American populations based on current knowledge. Using analysis of variance tests, frequency distribution was studied according to geographical, environmental, and cultural parameters. Frequencies were highest in Amazonian populations. In contrast, DI*A was undetectable in subarctic, Fuegian, Panamanian, Chaco and Yanomama populations. Closer study revealed a correlation that this unequal distribution was correlated with language, suggesting that linguistic divergence was a driving force in the expansion of DI*A among Native Americans. The absence of DI*A in circumpolar Eskimo-Aleut and Na-Dene speakers was consistent with a late migratory event confined to North America. Distribution of DI*A in subtropical areas indicated that gene and culture exchanges were more intense within than between ecozones. Bolstering the utility of classical genetic markers in biological anthropology, the present study of the expansion of Diego blood group genetic polymorphism in Native Americans shows strong evidence of gene-culture comigration.

## Introduction

Genetic diversity in Native Americans is an inexhaustible field of investigation. In the pre- molecular biology era, protein, enzyme, and red cell antigen polymorphisms were extensively studied [1, 2]. An outstanding achievements using such classical genetic markers was demonstration of a biological continuum between Siberian and Native American populations [3] in concordance with archaeological, craniofacial and molecular similarities on either side of the Bering Strait [4].

The Diego blood group system was the first, and perhaps most convincing, genetic marker linking Amerindians to Siberia [5, 6]. It was discovered in 1953 in a Venezuelan woman who experienced three obstetric accidents. Study showed that severe hemolytic anemia after childbirth was due to isoimmunization caused by an irregular antibody. This antibody along with the targeted previously unknown antigen were designated as the Diego system: Di^a^, and anti-Di^a^ [7] using the woman’s married name. Since then, a total of 22 Diego antigens have been discovered dispersed at 16 antigenic sites on Band3. Further study has also erected the Di^a^/Di^b^ on the last extramembrane loop and indicated the null phenotype Di(a-b-) has never been observed. A single substitution (rs2285644C>T, Chr17:42328621) defines two alleles, DI*B and DI*A responsible for the Di^b^ and Di^a^ antigens. The substitution occurs in the *SLC4A1* gene (chromosome 17) which codes for the erythrocyte membrane glycoprotein band 3 [8, 9]. While total absence of band 3 is incompatible with life, an altered form involving another allele—a 27-base pair deletion [10]—results in the Southeast Asian ovalocytosis that confers some degree of protection against cerebral malaria due to *Plasmodium falciparum* and *vivax* [11].

After its discovery, anti-Diego serum was used for a worldwide screening survey to identify individuals whose red cells agglutinate (or not) in the presence of the Diego factor (i.e. Di^a^ antigen). Results showed that Di^b^ antigen was ubiquitous but that Di^a^ was exclusive to populations of Mongolian-descent from Poland to South America and totally absent in African, Aborigine and European populations [2, 7]. Another noteworthy finding was the lack of Di^a^ in some Native Americans. Subsequently, investigators proposed that high and low frequency of Di^a^ reflected two distinct migratory events in South America, i.e. one wave without Di^a^ followed by one with Di^a^ [12]. However, this theory failed to explain the absence of Di^a^ in Native American peoples living in the arctic regions that supposedly settled more recently [13–15].

In comparison with Eurasian and African populations, present-day Native Americans display lower genetic diversity and ubiquitous polymorphisms [16, 17]. These features have been attributed to serial founder effects during migration from Africa to the southern cone of America resulting from the small effective size of populations crossing the Bering Strait and passing the Panama Isthmus [18]. One of the most stunning consequences of this process is serological homogeneity at the ABO and RH level since Central to South Amerindians almost all belong to the O-positive blood type and display the world’s highest frequencies of the Mongolian-origin Di^a^ antigen [1]. Despite the anthropological interest of Diego blood group system in Native Amerindian populations, recent study has been confined mostly to ABO and immunoglobulin genetic polymorphisms [19, 20]. As a result, the predominance of Di^a^ in the Americas has remained unexplored [21]. In particular, little attention has been given to the paramount goal of biological anthropology, i.e., the extent to which genetic diversity mirrors the observed archaeological, linguistic, and culture [22].

This study was undertaken to determine the present-day distribution of the Di^a^ antigen in Native American populations. For this purpose we assumed that the geographic distribution of the DI*A allele coding for Di^a^ is not random but rather coincided with cultural traits according to the gene-culture comigration concept. Our goal was to know if areas of high and low DI*A allele frequency correlated with cultural and natural traits. More specifically, we sought to detect significant differences in DI*A allele frequency between populations of distinct linguistic families, climatic conditions and subsidence strategies and to identify the parameter most closely associated with allele variance.

## Material and Methods

### Data collection

By reviewing previous studies on Di^a^ antigen in North, Central and South America, we ascertained the mean frequency of the DI*A allele in 144 Native American populations. If only the number of Di(a+) phenotypes was mentioned so that it was not possible to distinguish homozygous from heterozygous, we estimated the frequency of the DI*A allele using Bernstein's gene-counting method with the formula *p*_DI*A_ = 1-√[1-f(Di(a+))], where f(Di(a+)) is the proportion of Di^a^ antigen carriers in the population [23]. By assuming Hardy-Weinberg equilibrium, this method allows calculation of gene frequencies from phenotype frequencies even in the presence of ambiguous cases (e.g. recessive). This approach prevents any further selection tests from those frequencies. It should also be noted that the method lacks reliability for genetic systems with more the three alleles [24].

The following cultural and environmental traits were collected for each of the 144 populations: linguistic family, subsistence strategy, crop landraces, climate conditions, and mosquito infestation (S1 File, S1 Table). As a basis for linguistic family assignment, we used Ruhlen's classification that divided the studied populations into 15 Native American language groups [25]. Pre-Columbian subsistence strategy was defined as hunter-gatherer/forager, marine hunter-gatherer, and agriculturist [26–28]. This 3-category breakdown is admittedly debatable since strategies surely changed over time and also because populations may have used several strategies or even switched back and forth on a seasonal basis. Subtropical Central and South America were subdivided into areas harboring wild taxa of either maize (*Zea* genus [29]) or cassava (*Manihot* genus) [30]. Lastly, ecological areas were defined according to the updated Köppen-Geiger climate classification [31] and consideration of eight Anopheline species [32].

### Depicting the geographical dispersal of DI*A

All DI*A allele frequency data were plotted onto a single map using the Kriging algorithm of SURFER software 8.0. Spatial autocorrelation analysis was performed with the GENALEX software [33]. Using this approach, it was possible to process genetic similarity in allele frequencies in function of geographic separation [34]. We were thusly able to depict the correlation coefficient (r) of DI*A frequency between pairs of populations showing geographic separation falling within a specified distance class.

### Analysis of variance

In order to detect a pattern of the DI*A allele frequency amongst geographical, cultural or ecological groups, we first ran an analysis of molecular variance (AMOVA) using ARLEQUIN software 3.5 [35]. To ensure symmetrical distribution of DI*A variance across the Americas, a preliminary AMOVA was performed assuming that the 144 Native American populations were a single group. Subsequent analyses tried to identify the highest proportion of variance among several pool groups according to S1 Table.

We then tried to determine whether culture explained a significant proportion of DI*A variance even when geography was held constant. This was done by considering geographic groups that showed significant proportion of variance of DI*A and running three procedures, i.e., a two-way ANOVA (S1 Fig), an ANOVA on geographical residuals scores, and mixed models with XLSTAT Version 2014.6.05. ANOVA models were evaluated using the Fisher F test to determine whether the amount of information provided by the selected factors was significant enough to explain the variation of DI*A allele frequency in comparison with a supposedly constant rudimentary model and correspond to mean DI*A allele frequency. The use of residuals scores allows testing of factors possibly underlying deviation of geographical residuals from normal distribution. Mixed models attempted to a linear explanation for quantitative variables based on factors associated with fixed and random effects [36]. Here we set geography and culture as fixed and random effects, and looked at the significance of Z, the matrix of the random effects.

### Supplemental analysis of genetic variation associated with *SLC4A1* locus

In order to distinguish patterns of variation amongst carriers of DI*A and DI*B, we looked for higher resolution genetic markers surrounding the Diego locus. Assuming that variation could have been driven alongside *SLC4A1*, we reappraised the variation at 8 autosomal microsatellites (STRs) around *SLC4A1* in 28 Native American populations previously screened for 678 genome-wide STRs [37]. Genetic markers are D17S1294, GATA169F02, GGAA19G04, D17S1299, D17S2180, AAT245_17, and D17S1290678. They are embedded in a 20Mb-wide window on both sides of *SLC4A1*. Information can be found in the Mammalian Genotyping Marshfield Screening Sets 16 and 54 (http://research.marshfieldclinic.org/genetics/). We ran STRUCTURE [38] based on the admixture model from K = 2 to K = 10 with a burn-in period of 20,000 iterations followed by 10,000 iterations and displayed the results using DISTRUCT 1.1 [39] (S2 Fig).

## Results

**Fig 1:**
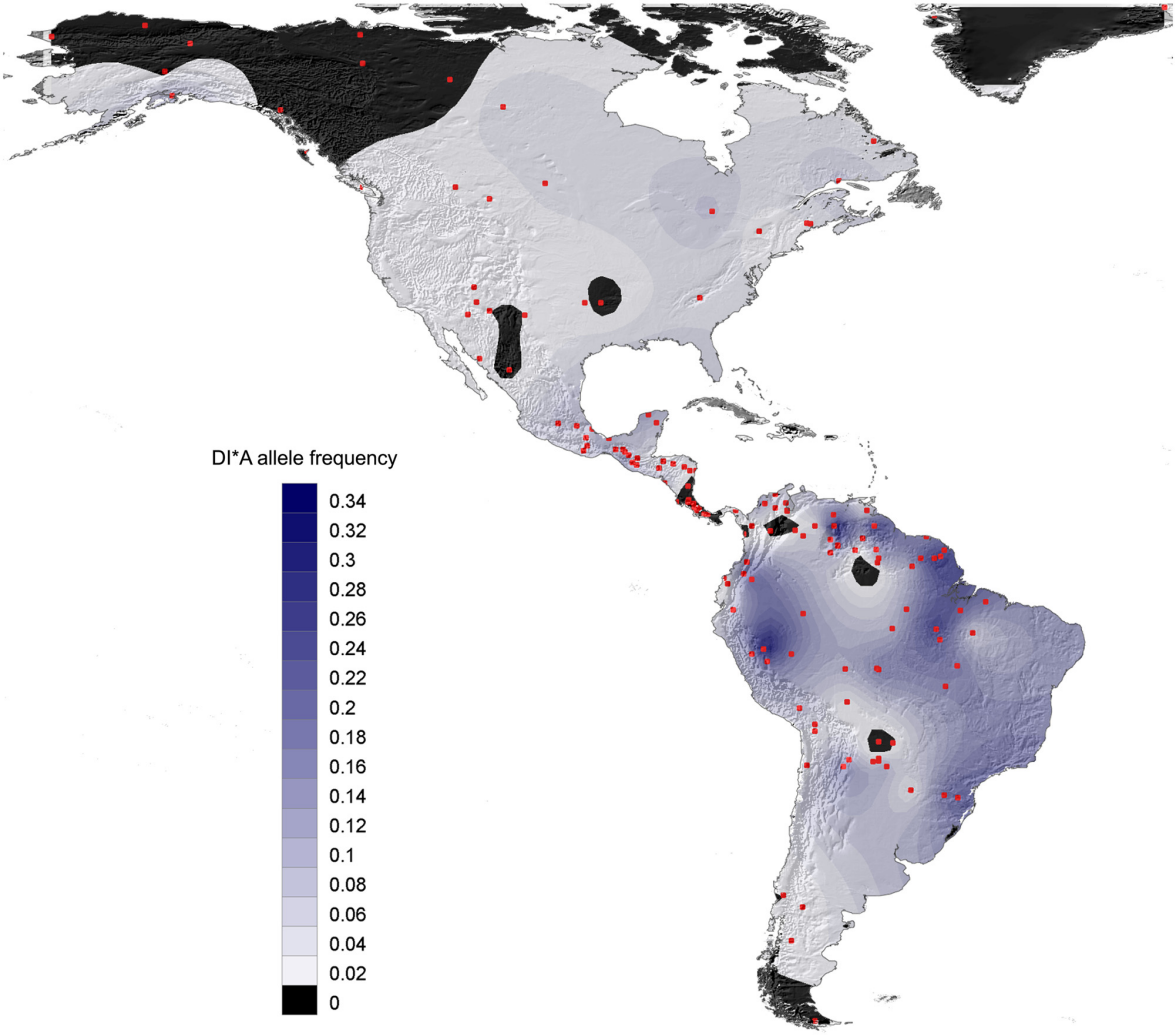
Mapping of DI*A allele frequency amongst 144 North, Central and South Amerindians.

**Fig 2:**
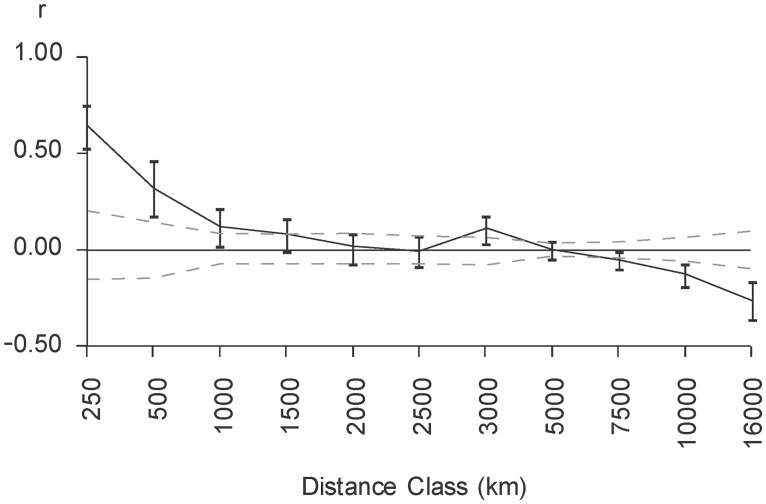
Spatial pattern of correlation of DI*A allele frequencies. Dotted lines indicate a 95% probability interval obtained with 1000 permutations for a null hypothesis of no spatial structure.

Fig 1 shows the highly contrasting geographic distribution of DI*A allele frequency in the Americas based on study of 144 Native American populations. High frequencies were observed in the Peruvian Andes, on the Guyanese Plateau, in the southeastern Amazonian Basin, in a region comprising the Brazilian state of Pará, and along the Tapajos, Xingu and Araguaia tributaries of the Amazon River. Low frequencies occurred mainly in North America, in Chaco and at southern tip of South America. The DI*A allele was totally absent in the Arctic (Eskimo and Tlingit), Panama Isthmus (Bribri and Teribe), Tierra de Fuego (Alacalufe), and a few pocket areas in North America (Cherokee), Northern Brazil/Southern Venezuela (Ninam and Yanomama), and the Chaco area (Ayoreo, Zamucoan). Fig 2 summarizes analysis of the correlation between differences in DI*A frequency and geographic distance in paired populations. The observed pattern refutes the null correlation hypothesis (p<0.001) by showing an increasingly significant positive correlation as the distance between populations decrease. This finding suggests the existence of locations with similar genetic features, i.e., where populations have similar DI*A allele frequencies.

When the Diego polymorphism (S1 Table) was portrayed in relation to language, highest DI*A frequencies were observed for the Equatorial-Tucanoan and Ge-Pano-Karib branches of the Southern Amerind linguistic family, particularly among Tucanoan speakers (mean frequency = 0.229), Panoan (0.223), Karib (0.194), Tupi (0.184), and Ge (0.171). The DI*A allele was almost completely absent (less than 2%) in Eskimo-Aleut, Na-Dene, and Keresiouan speakers from North America and Chibchan speakers from Lower Mesoamerica. Portrayal of polymorphism in relation to subsistence mode and ecological conditions indicated that DI*A was more frequent in hunter-gatherer/forager (mean frequency = 0.133) than in farmer (0.061) and marine hunter/gatherer (0.018) groups. Equatorial regions accounted for up to 12% of DI*A and frequencies decreased in gradient fashion from warm temperate (mean = 0.076) to arid (0.045), snowy (0.032) and polar (0.000) regions. A higher frequency of DI*A was observed in populations living in areas where the domesticated crop was cassava (0.176) rather than maize (0.081) or neither (0.039).

In the second phase of study particular attention was paid to determine if areas with similar DI*A allele frequencies occurred randomly or rather coincided with geography, linguistics, lifestyle, or ecoregions. Table 1 presents the percentage of DI*A allele frequency variation obtained using various one-way models to identify the factor most closely associated with allele variance. Overall, the Diego blood group exhibited a significant variation in the Americas accounting for 11.95% of the total frequency variance between the 144 Native American populations. Geography accounted for significant between- group allele variation (2.77%, p = 0.043; 3.24%, p = 0.006). Grouping according to the three main linguistic phyla, i.e. Eskimo-Aleut, Na-Dene, and Amerind, failed to reveal a significant genetic structure for the system of interest (variation, 3.54%, p>0.05) whereas grouping according to Native American linguistic family as defined by Ruhlen was significant (variation, 4.71%, p = 0.001). Lifestyle accounted for 3.61% of DI*A allele frequency variation among Amerindians, with highest values being reached whether the populations inhabited areas with wild taxa of cassava or not (variation, 9.59% and 13.36%, p = 0.000). Grouping according to putative mode of subsistence and alimentary crop in relation to maize showed little (Zea species: p = 0.029) and no significant pattern of variance of DI*A (presence/absence: p = 0.489). Climate did not account for the observed DI*A allele differences (variation, 1.23%, p = 0.257 and 2.13%, p = 0.152) but ecological areas defined by anopheles mosquito was correlated with DI*A allele frequency (variation range, 7.85% to 12.38%, p = 0.000).

**Table 1:** Proportion of variance of DI*A allele frequency in 144 Native American populations.

Groups		N° of groups	Within groups (p-value)	Among groups (p-value)
None		1	11.95 (0.000)	
Geography	North/Central/South America	3	10.09 (0.000)	3.24 (0.006)
	Subarctic/North/Central/Lower Meso/Southwestern/Andean/Chaco/Eastern	8	9.57 (0.000)	2.77 (0.043)
Linguistic	Eskimo-Aleut/Na-Dene/Amerind	3	11.05 (0.000)	3.54 (0.077)
	Ruhlen's classification	15	7.68 (0.000)	4.71 (0.001)
Mode of subsistence	Humanized landscape	2	10.91 (0.000)	3.62 (0.045)
	Agriculturist/Hunter-gatherer/Marine hunter-gatherer	3	9.80 (0.000)	3.61 (0.008)
	Presence/Absence of Zea	2	12.04 (0.000)	-0.22 (0.489)
	Zea species	8	1.94 (0.000)	0.43 (0.029)
	Presence/Absence of Manihot	2	5.27 (0.000)	13.36 (0.000)
	Zea species/presence of Manihot/none	9	5.41 (0.000)	9.59 (0.000)
Environmental centers	Equatorial/arid/warm temperature/snow polar	5	11.34 (0.000)	1.23 (0.257)
	Climate and precipitation	11	10.22 (0.000)	2.13 (0.152)
	*A*.*nuneztovari-albitarsis-darling-freeborni*/none	2	5.98 (0.000)	12.38 (0.000)
	Anopheles species	5	6.33 (0.000)	7.85 (0.000)

In the third phase of study, we tested the gene-culture concept with respect to the effects of four cultural traits in the dispersal of DI*A amongst geographically determined populations. Table 2 presents the results obtained by ANOVA and mixed models using four cultural traits and two geographic divisions, and S1 Fig the interactions. When controlled for three main regions ANOVAs showed significant effects of languages, lifestyle, and crop type (Fisher's p value range: 0.000–0.013, Figures A, B and C in S1 Fig). However, lifestyle and combined maize and cassava crop type barely reached significance (Fisher's p value = 0.041) or even failed to explain abnormal distribution of the geographical residuals (Fisher's p values: 0.071; 0.0193, Figure D in S1 Fig). Lifestyle, cassava crop type, and combined maize and cassava crop type failed to linearize the DI*A allele frequency. All tests indicated that language was the most explanatory factor (p-value range: 0.000–0.036).

**Table 2:** ANOVAs of DI*A allele frequency in 144 Native American populations after removal of geography effects. Df: degree of freedom. Fisher's F: test of the amount of information provided by the selected factors in comparison with the mean DI*A allele frequency. Z: Random effects.

Factor		Three regions hold constant			Eight regions hold constant		
		Two-way	Geography residuals	Mixed linear model	Two-way	Geography residuals	Mixed linear model
	Df	F (p value)	F (p value)	Z (p value)	F (p value)	F (p value)	Z (p value)
Ruhlen's linguistic classification	14	3,596 (0.000)	3,204 (0.000)	1,794 (0,036)	4,145 (0.000)	2,987 (0,001)	1,817 (0,035)
Life Style	2	6,779 (0,002)	4,484 (0.013)	0,835 (0,202)	3,280 (0.041)	1,665 (0,193)	0,690 (0,245)
Presence of Manihot	1	37,869 (0.000)	25,324 (0.000)	0,688 (0,246)	16,152 (0.000)	7,121 (0,009)	0,663 (0,254)
Zea species/ areas of Manihot/none	8	6,671 (0.000)	5,609 (0.000)	1,547 (0,061)	3,236 (0.002)	1,860 (0,071)	1,282 (0,100)

Lastly, we tried to determine if highly polymorphic genetic markers in the vicinity of the *SLC4A1* locus could help understand the observed variation (S2 Fig). Except with regard to exclusion of the Aché, Surui, Kogi, Pima, and a few Waunana individuals, the bar plots do not show any peculiar genetic structure.

## Discussion

Six decades ago, demonstration that the Di^a^ antigen was a shared red cell feature of most Native American and Asian populations served as proof that present-day North, Central and South Amerindians originated from Siberia. Despite its significant anthropological interest, the causative factors underlying the current distribution of Di^a^ in Native Americans populations has remained unclear [21]. This study was designed to gain insight into this aspect by correlating reported data on the frequency of the allele coding for Di^a^ (DI*A) with environmental and cultural factors. Our assumption was that expansion of the Diego blood group from North to South America was also driven by culture.

### Diego reflects the cultural divergence of South Native Americans

Whether or not human genetic diversity occurred randomly is a fundamental question in the field of biological anthropology. Mapping genetic variation amongst populations has proven to be a useful technique to identify meaningful concordances with historic populations or ecological niches [2, 40]. The data on reported here indicate that DI*A allele frequencies show striking contrasts between areas and that variations tend to correlate significantly with environmental and cultural traits. The strongest correlations were observed in the Amazon region as opposed to the rest of the continent and in function of linguistic family. Analysis according to linguistic family revealed a significant correlation between with the phylogenetic structure of the Amerind language family with high DI*A levels in Southern Amerind language speakers. High correlation with cassava and Tupi-speaking areas agrees with expansion driven by agriculture [41]. The current genetic structure of the Diego blood group may mirror the congruent divergence mechanisms that have shaped the South Amerindian genetic and linguistic diversity of Andean, Lower Meso, and Meso Amerindians [37, 42, 43]. Observation of significant genetic kinship between geographically proximate linguistically similar groups has been interpreted as evidence of North-to-South population migration followed by divergence resulting in genetic and linguistic variation during the complex demographic history of the Andes and Amazon Basin [37, 44, 45].

Another striking finding of the present study involves areas with low DI*A levels. Local genetic differentiation associated high genetic drift and uneven gene flow could explain this finding especially in the Yanomama populations [46] who provide an outstanding example for gene-culture modeling. Villages form due to social or political tensions as dissident groups of related individuals broke off from the main population and settled in nearby locations where they possibly mixed with other people before returning to the original population. This process involves several neighboring villages and recurs over generations. Fission-fusion is a formidable enhancer of genetic differentiation and may explain the differences in peripheral genetic behavior between the Yanomama and surrounding populations [47–49].

Previous studies have described the special genetic and craniometric characteristics of the Ayoreo (a.k.a. Moro) in the Chaco region. Like the Aché, Emerillon, and Yaghan people, the Ayoreo exhibit limited within-population diversity that is among the lowest in South America. These genetic peculiarities are most likely due to a severe founder effect, but the exact causative events remain unclear [50, 51].

In the Isthmus of Panama, craniofacial, classical and molecular investigations have documented the genetic distinctiveness of present-day Chibchan-speakers suggesting arguing that their predecessors have lived in isolation from Central and South Amerindians since the early Holocene [37, 52, 53]. It is noteworthy that Chibchan-speakers are thought to be latecomers to cultivation of maize and manioc [53]. Hence, the Isthmus of Panama could represent a nexus for the southward expansion of maize cultivation and northward spread of arrowroot and manioc cultivation without gene flows probably since 7500 cal BP [54, 55]. The lack of Di^a^ antigen in the Isthmus of Panama would be consistent with the long-term preservation of the Chibchan genetic substratum.

### Did comigration act alone?

Based on dispersal patterns coupled with the demographic history of Native Amerindians, it is not unexpected to see a loss of genetic diversity in most of the Eastern South Amerindians [18, 44, 45, 56]. Herein, parceling out of DI*A amongst South Amerindians reinforced by spatial autocorrelation analysis suggests that isolation by distance and genetic drift in Eastern South Amerindians populations also played a non-negligible role. These factors may have led to random accumulation or depletion of DI*A in the same way as for private polymorphisms [46, 57]. This process is highly likely and could have occurred concomitantly with the above-mentioned population expansion.

Our study also revealed a significant correlation between DI*A allele frequency and warm tropical conditions, domesticated crop type, and presence of disease-carrying vector species. The circumscribed areas defined by these factors compose a mosaic of specific biocenoses and pathocenoses [31, 32]. It is thus reasonable to consider natural selection in the distribution of human genetic polymorphisms [58]. However, testing for selection was not possible with the frequency data and no pattern could be drawn from the reappraised autosomal microsatellites—certainly because of their distance from *SLC4A1* (the closest being D17S2180 at >1.6 Mb). An alternative explanation for the correlation with tropical conditions is that ecology may have contributed to higher gene flow rates within a similar environment and induced distortion in the distribution of the DI*A allele frequency between distinct ecological centers [44]. Analysis of spatial autocorrelation and systematic observation of significant correlation with subtropical conditions support greater inter-ethnic exchanges within than between ecological environments [44].

### Insights into peopling of the Americas

Unraveling the genetic structure of Native Americans holds the key to depicting the patterns of the initial peopling of the Americas. Deeper screening of the variation of the ABO-RH blood group, classical genetic markers, or Y-chromosome failed to show clear-cut genetic patterns amongst the O+ phenotypes or Q-M3 male lineages [59, 60]. Two likely explanations involve either sample bias preventing accurate evaluation of the distribution of these sublineages or insufficient sampling not covering enough populations to allow tracing back genetic relationships at the continental level [17, 19, 61]. Another highly plausible reason is that most North, Central and South Amerindians have a common ancestry and thus share common genetic features throughout the Americas [16, 62–65]. Our results concord with previous screening of high-resolution genetic markers that demonstrated genetic similarities in Native Americans populations in correlation with shared geographical, linguistic and even demographic background [37, 42, 45, 59, 66, 67].

Absence of DI*A in the northernmost and southernmost regions of the American continent is another noteworthy finding. While isolation by distance with ensuing drift likely accounts for genetic variation in Fuegians [26], an alternative interpretation can be proposed for the present-day gene pool around Beringia. It is that additional gene flows occurred as new populations continued to cross over and spread eastward and southward after the initial migratory wave that supposedly colonized the Americas 15–20 ky ago [3, 42]. Secondary migration would account for the presence of non-Amerind-speakers, i.e., Eskimo-Aleut and Na-Dene speakers, from Alaska to Greenland and Southwestern North America. Though still controversial [68], reverse-migration of Beringian populations back into Siberia is also thought to have occurred [66], thereby explaining the relationship observed between the Yeniseian languages in Central Siberia and Na-Dene languages in North America [69, 70]. Interestingly, absence of DI*A is a shared characteristic of Eskimo-Aleut, Na-Dene, and Yeniseian-speakers [2]. Our results are compatible with the hypothesis that an additional gene flow involving mostly DI*B allele carriers occurred in the northernmost areas of North America.

Whether or not it is realistic to attempt to reconstruct the genetic history of peopling from a single locus is pertinent question. It is difficult to ascertain that deviation of allele frequency is due to population displacement only and not to genetic drift [59, 60]. Paucity in encapsulating the genetic variability also weakens the inferences. Fortunately, study of single genetic markers has furnished relevant insight into population ancestry, genetic relationships and migration. Blood group systems [16, 20, 71] and haplogroup-specific works [72–74], that involve study of expansion of no more than one lineage of SNPs, are devoted in this purpose. Uniparental loci are drift-sensitive but gain in accuracy when enriched with highly polymorphic linked genetic markers. Herein we assumed that DI*A and DI*B were sufficiently informative for preliminary study of Native American population dispersal. In this era of fine-grain genetic screening [42], additional experiments on DI*A carriers will be needed to confirm our approach.

## Conclusion

The aim of this study was to assess the geographical distribution of the DI*A allele of the Diego blood group system in Amerindians. Our data demonstrates large variations in frequency ranges and a significant correlation with environmental and cultural parameters. Our findings suggest that DI*A carriers crossed into Americas from Siberia and have diverged along the lines of the main linguistic families. Subsequent genetic differentiation appears to have been stronger in Lower Meso and South Amerindians depending on environment conditions. In the Isthmus of Panama, Chibchan-speakers appear to have experienced intense cultural interactions with neighboring populations without relevant gene flow. A secondary migration by mostly DI*B carriers may have covered North America. In addition to providing strong evidence of gene-culture comigration in the Americas, the present study illustrates the extent to which geographical maps of genetic and cultural phenomena dovetail.

## Supporting Information

S1 File Readme file for S1 Table. (DOC)

S1 Table Distribution of the 144 populations considered in the present study according to geographic, cultural and environmental parameters, listed according to latitude. The file is a MS Excel spreadsheet 97–2003 (.xls) and is coupled with a Readme file (S1 File).
(XLS)

S1 Fig Interactions between culture and geography from the two-way ANOVA of DI*A allele frequencies (Table 2). Left panels: three main areas where controlled. Right panels: eight main areas where controlled. Ruhlen's linguistic classes (Figure A). Main Pre-Colombian subsidence strategies (Figure B). Areas with/without cassava (Figure C). Areas with/without maize or cassava crops (Figure D). See S1 Table and S1 File for complete references.
(TIF)

S2 Fig Unsupervised genetic structure of 28 Native American populations based on 8 autosomal STRs (D17S1294, GATA169F02, GGAA19G04, D17S1299, D17S2180, AAT245_17, and D17S1290) surrounding the *SLC4A1* locus (chromosome 17). Samples were genotyped by [37].
(TIF)

## References

[1] Mourant AE, Kopec AC, Domaniewska-Sobczak K. The ABO blood groups: Comprehensive tables and maps of world distribution. Blackwell Scientific Publications ed. Oxford: Blackwell Scientific Publications; 1958 276 p.

[2] Cavalli-Sforza LL, Menozzi P, Piazza A. The history and geography of human genes. Press PU, editor. Princeton: Princeton University Press; 1994.

[3] Williams RC, Steinberg AG, Gershowitz H, Bennett PH, Knowler WC, Pettitt DJ, et al GM allotypes in Native Americans: evidence for three distinct migrations across the Bering land bridge. Am J Phys Anthropol. 1985;66(1):1–19. 10.1002/ajpa.1330660102 . 3976868

[4] Goebel T, Waters MR, Dikova M. The archaeology of Ushki Lake, Kamchatka, and the Pleistocene peopling of the Americas. Science. 2003;301(5632):501–5. . 12881567 10.1126/science.1086555

[5] Levine P, Robinson EA, Layrisse M, Arends T, Domingues Sisco R. The Diego blood factor. Nature. 1956;177(4497):40–1. . 13288586 10.1038/177040b0

[6] Layrisse M, Arends T. The Diego blood factor distribution; genetic, clinical and anthropological significance. Bibl Haematol. 1958;7:114–6. . 13499239 10.1159/000427069

[7] Junqueira PC, Castilho L. The history of the Diego blood group. Rev Bars Hematol Hemoter. 2002;24(1):15–23.

[8] Storry JR, Castilho L, Daniels G, Flegel WA, Garratty G, Francis CL, et al International Society of Blood Transfusion Working Party on red cell immunogenetics and blood group terminology: Berlin report. Vox Sang. 2011;101(1):77–82. 10.1111/j.1423-0410.2010.01462.x . 21401621 PMC5497848

[9] Spring FA, Bruce LJ, Anstee DJ, Tanner MJ. A red cell band 3 variant with altered stilbene disulphonate binding is associated with the Diego (Dia) blood group antigen. Biochem J. 1992;288 (Pt 3):713–6. . 1471983 10.1042/bj2880713 PMC1131943

[10] Jarolim P, Palek J, Amato D, Hassan K, Sapak P, Nurse GT, et al Deletion in erythrocyte band 3 gene in malaria-resistant Southeast Asian ovalocytosis. Proc Natl Acad Sci U S A. 1991;88(24):11022–6. . 1722314 10.1073/pnas.88.24.11022 PMC53065

[11] Booth PB, Serjeantson S, Woodfield DG, Amato D. Selective depression of blood group antigens associated with hereditary ovalocytosis among melanesians. Vox Sang. 1977;32(2):99–110. . 403680 10.1111/j.1423-0410.1977.tb00612.x

[12] Layrisse M, Wilbert J. Absence of the Diego antigen, a genetic characteristic of early immigrants to South America. Science. 1961;134:1077–8. . 14463057 10.1126/science.134.3485.1077

[13] Gilbert MT, Kivisild T, Gronnow B, Andersen PK, Metspalu E, Reidla M, et al Paleo-Eskimo mtDNA genome reveals matrilineal discontinuity in Greenland. Science. 2008;320(5884):1787–9. 1159750 [pii] 10.1126/science.1159750 . 18511654

[14] Raghavan M, DeGiorgio M, Albrechtsen A, Moltke I, Skoglund P, Korneliussen TS, et al The genetic prehistory of the New World Arctic. Science. 2014;345(6200):1255832 345/6200/1255832 [pii] 10.1126/science.1255832 . 25170159

[15] Raghavan M, Skoglund P, Graf KE, Metspalu M, Albrechtsen A, Moltke I, et al Upper Palaeolithic Siberian genome reveals dual ancestry of Native Americans. Nature. 2014;505(7481):87–91. nature12736 [pii] 10.1038/nature12736 . 24256729 PMC4105016

[16] Schroeder KB, Schurr TG, Long JC, Rosenberg NA, Crawford MH, Tarskaia LA, et al A private allele ubiquitous in the Americas. Biol Lett. 2007;3(2):218–23. J1210G233485MM4M [pii] 10.1098/rsbl.2006.0609 . 17301009 PMC2375964

[17] Battaglia V, Grugni V, Perego UA, Angerhofer N, Gomez-Palmieri JE, Woodward SR, et al The first peopling of South America: new evidence from Y-chromosome haplogroup Q. PLoS One. 2013;8(8):e71390 10.1371/journal.pone.0071390 PONE-D-13-19844 [pii]. . 23990949 PMC3749222

[18] Henn BM, Cavalli-Sforza LL, Feldman MW. The great human expansion. Proc Natl Acad Sci U S A. 2012;109(44):17758–64. 1212380109 [pii] 10.1073/pnas.1212380109 . 23077256 PMC3497766

[19] Estrada-Mena B, Estrada FJ, Ulloa-Arvizu R, Guido M, Mendez R, Coral R, et al Blood group O alleles in Native Americans: implications in the peopling of the Americas. Am J Phys Anthropol. 2010;142(1):85–94. 10.1002/ajpa.21204 . 19862808

[20] Dugoujon JM, Hazout S, Loirat F, Mourrieras B, Crouau-Roy B, Sanchez-Mazas A. GM haplotype diversity of 82 populations over the world suggests a centrifugal model of human migrations. Am J Phys Anthropol. 2004;125(2):175–92. 10.1002/ajpa.10405 . 15365983

[21] Anstee DJ. The relationship between blood groups and disease. Blood. 2010;115(23):4635–43. blood-2010-01-261859 [pii] 10.1182/blood-2010-01-261859 . 20308598

[22] Laland KN, Odling-Smee J, Myles S. How culture shaped the human genome: bringing genetics and the human sciences together. Nat Rev Genet. 2010;11(2):137–48. nrg2734 [pii] 10.1038/nrg2734 . 20084086

[23] Bernstein F. Zusammenfassende Betrachtungen uber die erblichen Blutstrukturen des Menschen. Zeitschrift für induktive Abstammungs- und Vererbungslehre. 1925;37:237–370.

[24] Nam JM, Gart JJ. Bernstein's and gene-counting methods in generalized ABO-like systems. Ann Hum Genet. 1976;39(3):361–73. . 1275447 10.1111/j.1469-1809.1976.tb00141.x

[25] Ruhlen M. A Guide to the World’s Languages, Vol. 1: Classification. Press SSU, editor1987.

[26] Gonzalez-Jose R, Garcia-Moro C, Dahinten S, Hernandez M. Origin of Fueguian-Patagonians: an approach to population history and structure using R matrix and matrix permutation methods. Am J Hum Biol. 2002;14(3):308–20. 12001087 10.1002/ajhb.10033

[27] Hunemeier T, Amorim CE, Azevedo S, Contini V, Acuna-Alonzo V, Rothhammer F, et al Evolutionary responses to a constructed niche: ancient Mesoamericans as a model of gene-culture coevolution. PLoS One. 2012;7(6):e38862 10.1371/journal.pone.0038862 PONE-D-12-04340 [pii]. . 22768049 PMC3380856

[28] Steward JH, editor. Handbook of South American Indians—Tome I, II, III, V, and VI. New York: Cooper Square; 1963.

[29] Matsuoka Y, Mitchell SE, Kresovich S, Goodman M, Doebley J. Microsatellites in Zea—variability, patterns of mutations, and use for evolutionary studies. Theor Appl Genet. 2002;104(2–3):436–50. 10.1007/s001220100694 . 12582717

[30] Leotard G, Duputie A, Kjellberg F, Douzery EJ, Debain C, de Granville JJ, et al Phylogeography and the origin of cassava: new insights from the northern rim of the Amazonian basin. Mol Phylogenet Evol. 2009;53(1):329–34. S1055-7903(09)00176-6 [pii] 10.1016/j.ympev.2009.05.003 . 19435608

[31] Kottek M, Grieser J, Beck C, Rudolf B, Rubel F. World Map of the Köppen-Geiger climate classification updated. Meteorologische Zeitschrift. 2006;15(3):259–63.

[32] Sinka ME, Rubio-Palis Y, Manguin S, Patil AP, Temperley WH, Gething PW, et al The dominant Anopheles vectors of human malaria in the Americas: occurrence data, distribution maps and bionomic precis. Parasit Vectors. 2010;3:72 1756-3305-3-72 [pii] 10.1186/1756-3305-3-72 . 20712879 PMC2936890

[33] Peakall R, Smouse PE. GenAlEx 6.5: genetic analysis in Excel. Population genetic software for teaching and research—an update. Bioinformatics. 2012;28(19):2537–9. bts460 [pii] 10.1093/bioinformatics/bts460 . 22820204 PMC3463245

[34] Slatkin M, Arter HE. Spatial autocorrelation methods in population genetics Am Nat. 1991;138:499–517.

[35] Excoffier L, Laval G, Schneider S. Arlequin (version 3.0): An integrated software package for population genetics data analysis. Evolutionary bioinformatics online. 2005;1:47–50. . 19325852 PMC2658868

[36] Wolfinger RD. Covariance structure selection in general mixed models. Communications in Statistics, Simulation and Computation. 1993;22(4):1079–106.

[37] Wang S, Lewis CM, Jakobsson M, Ramachandran S, Ray N, Bedoya G, et al Genetic variation and population structure in native Americans. PLoS Genet. 2007;3(11):e185 . 18039031 10.1371/journal.pgen.0030185 PMC2082466

[38] Pritchard JK, Stephens M, Donnelly P. Inference of population structure using multilocus genotype data. Genetics. 2000;155(2):945–59. . 10835412 10.1093/genetics/155.2.945 PMC1461096

[39] Rosenberg NA. DISTRUCT: a program for the graphical display of population structure. Mol Ecol Notes. 2004;4:137–8.

[40] Rosenberg NA, Pritchard JK, Weber JL, Cann HM, Kidd KK, Zhivotovsky LA, et al Genetic structure of human populations. Science. 2002;298(5602):2381–5. 10.1126/science.1078311 298/5602/2381 [pii]. . 12493913

[41] Epps P. Language Classification, Language Contact, and Amazonian Prehistory. Language and Linguistics Compass. 2009;3(2):581–606.

[42] Reich D, Patterson N, Campbell D, Tandon A, Mazieres S, Ray N, et al Reconstructing Native American population history. Nature. 2012;488(7411):370–4. nature11258 [pii] 10.1038/nature11258 . 22801491 PMC3615710

[43] Salzano FM, Hutz MH, Salamoni SP, Rohr P, Callegari-Jacques SM. Genetic Support for Proposed Patterns of Relationship among Lowland South American Languages. Current Anthropology. 2005;46:121–8.

[44] Tarazona-Santos E, Carvalho-Silva DR, Pettener D, Luiselli D, De Stefano GF, Labarga CM, et al Genetic differentiation in South Amerindians is related to environmental and cultural diversity: evidence from the Y chromosome. Am J Hum Genet. 2001;68(6):1485–96. S0002-9297(07)61059-3 [pii] 10.1086/320601 . 11353402 PMC1226135

[45] Yang NN, Mazieres S, Bravi C, Ray N, Wang S, Burley MW, et al Contrasting patterns of nuclear and mtDNA diversity in Native American populations. Ann Hum Genet. 2010;74(6):525–38. 10.1111/j.1469-1809.2010.00608.x . 20887376

[46] Neel JV, Gershowitz H, Spielman RS, Migliazza EC, Salzano FM, Oliver WJ. Genetic studies of the Macushi and Wapishana Indians. II. Data on 12 genetic polymorphisms of the red cell and serum proteins: gene flor between the tribes. Hum Genet. 1977;37(2):207–19. 407146 10.1007/BF00393584

[47] Thompson EA. Fission models of population variability. Genetics. 1979;93(2):479–95. . 535728 10.1093/genetics/93.2.479 PMC1214094

[48] Smouse PE, Vitzthum VJ, Neel JV. The impact of random and lineal fission of the genetic divergence of small human groups: a case study among the Yanomama. Genetics. 1981;98(1):179–97. . 7338303 10.1093/genetics/98.1.179 PMC1214427

[49] Hunley KL, Spence JE, Merriwether DA. The impact of group fissions on genetic structure in Native South America and implications for human evolution. Am J Phys Anthropol. 2008;135(2):195–205. 10.1002/ajpa.20720 . 18046773

[50] Dornelles CL, Battilana J, Fagundes NJ, Freitas LB, Bonatto SL, Salzano FM. Mitochondrial DNA and Alu insertions in a genetically peculiar population: the Ayoreo Indians of Bolivia and Paraguay. Am J Hum Biol. 2004;16(4):479–88. 15214066 10.1002/ajhb.20038

[51] Salzano FM, Pages F, Neel JV, Gershowitz H, Tanis RJ, Moreno R, et al Unusual blood genetic characteristics among the Ayoreo Indians of Bolivia and Paraguay. Hum Biol. 1978;50(2):121–36. . 689642

[52] Galland M. Le premier peuplement des Amériques: application de la morphométrie géométrique 3D à la variation crânienne actuelle et fossile. Paris: Muséum National d'Histoire Naturelle; 2013.

[53] Melton PE. Genetic history and pre-Columbian Diaspora of Chibchan speaking populations: Molecular genetic evidence. University of Kansas. Lawrence, KS: University of Kansas; 2008.

[54] Dickau R, Ranere AJ, Cooke RG. Starch grain evidence for the preceramic dispersals of maize and root crops into tropical dry and humid forests of Panama. Proc Natl Acad Sci U S A. 2007;104(9):3651–6. 0611605104 [pii] 10.1073/pnas.0611605104 . 17360697 PMC1805539

[55] Scliar MO, Gouveia MH, Benazzo A, Ghirotto S, Fagundes NJ, Leal TP, et al Bayesian inferences suggest that Amazon Yunga Natives diverged from Andeans less than 5000 ybp: implications for South American prehistory. BMC Evol Biol. 2014;14(1):174 s12862-014-0174-3 [pii] 10.1186/s12862-014-0174-3 . 25266366 PMC4189748

[56] Chiaroni J, Underhill PA, Cavalli-Sforza LL. Y chromosome diversity, human expansion, drift, and cultural evolution. Proc Natl Acad Sci U S A. 2009;106(48):20174–9. 0910803106 [pii] 10.1073/pnas.0910803106 . 19920170 PMC2787129

[57] Neel JV, Salzano FM. Further studies on the Xavante Indians. X. Some hypotheses-generalizations resulting from these studies. Am J Hum Genet. 1967;19(4):554–74. . 6036274 PMC1706315

[58] Fumagalli M, Sironi M, Pozzoli U, Ferrer-Admetlla A, Pattini L, Nielsen R. Signatures of environmental genetic adaptation pinpoint pathogens as the main selective pressure through human evolution. PLoS Genet. 2011;7(11):e1002355 10.1371/journal.pgen.1002355 PGENETICS-D-11-00857 [pii]. . 22072984 PMC3207877

[59] Bisso-Machado R, Bortolini MC, Salzano FM. Uniparental genetic markers in South Amerindians. Genet Mol Biol. 2012;35(2):365–87. 10.1590/S1415-47572012005000027 gmb-35-2-365 [pii]. . 22888284 PMC3389523

[60] Salzano FM. Molecular variability in Amerindians: widespread but uneven information. An Acad Bras Cienc. 2002;74(2):223–63. 12098752 10.1590/s0001-37652002000200005

[61] Perego UA, Angerhofer N, Pala M, Olivieri A, Lancioni H, Hooshiar Kashani B, et al The initial peopling of the Americas: a growing number of founding mitochondrial genomes from Beringia. Genome Res. 2010;20(9):1174–9. gr.109231.110 [pii] 10.1101/gr.109231.110 . 20587512 PMC2928495

[62] Bonatto SL, Salzano FM. A single and early migration for the peopling of the Americas supported by mitochondrial DNA sequence data. Proc Natl Acad Sci U S A. 1997;94(5):1866–71. 9050871 10.1073/pnas.94.5.1866 PMC20009

[63] Fagundes NJ, Kanitz R, Eckert R, Valls AC, Bogo MR, Salzano FM, et al Mitochondrial population genomics supports a single pre-Clovis origin with a coastal route for the peopling of the Americas. Am J Hum Genet. 2008;82(3):583–92. S0002-9297(08)00139-0 [pii] 10.1016/j.ajhg.2007.11.013 . 18313026 PMC2427228

[64] Underhill PA, Jin L, Zemans R, Oefner PJ, Cavalli-Sforza LL. A pre-Columbian Y chromosome-specific transition and its implications for human evolutionary history. Proc Natl Acad Sci U S A. 1996;93(1):196–200. 8552603 10.1073/pnas.93.1.196 PMC40205

[65] Villanea FA, Bolnick DA, Monroe C, Worl R, Cambra R, Leventhal A, et al Brief communication: Evolution of a specific O allele (O1vG542A) supports unique ancestry of Native Americans. Am J Phys Anthropol. 2013;151(4):649–57. 10.1002/ajpa.22292 . 23868176

[66] Tamm E, Kivisild T, Reidla M, Metspalu M, Smith DG, Mulligan CJ, et al Beringian standstill and spread of Native American founders. PLoS One. 2007;2(9):e829 . 17786201 10.1371/journal.pone.0000829 PMC1952074

[67] Regueiro M, Alvarez J, Rowold D, Herrera RJ. On the origins, rapid expansion and genetic diversity of Native Americans from hunting-gatherers to agriculturalists. Am J Phys Anthropol. 2013;150(3):333–48. 10.1002/ajpa.22207 . 23283701

[68] Rubicz R, Melvin KL, Crawford MH. Genetic evidence for the phylogenetic relationship between Na-Dene and Yeniseian speakers. Hum Biol. 2002;74(6):743–60. . 12617487 10.1353/hub.2003.0011

[69] Diamond J. Deep relationships between languages. Nature. 2011;476:291–2. 10.1038/476291a 21850102

[70] Sicoli MA, Holton G. Linguistic phylogenies support back-migration from beringia to Asia. PLoS One. 2014;9(3):e91722 10.1371/journal.pone.0091722 PONE-D-13-33587 [pii]. . 24621925 PMC3951421

[71] Koda Y, Tachida H, Soejima M, Takenaka O, Kimura H. Ancient origin of the null allele se(428) of the human ABO-secretor locus (FUT2). J Mol Evol. 2000;50(3):243–8. 10.1007/s002399910028 [pii]. . 10754067

[72] Rootsi S, Magri C, Kivisild T, Benuzzi G, Help H, Bermisheva M, et al Phylogeography of Y-chromosome haplogroup I reveals distinct domains of prehistoric gene flow in europe. Am J Hum Genet. 2004;75(1):128–37. 10.1086/422196 S0002-9297(07)62002-3 [pii]. . 15162323 PMC1181996

[73] Underhill PA, Poznik GD, Rootsi S, Jarve M, Lin AA, Wang J, et al The phylogenetic and geographic structure of Y-chromosome haplogroup R1a. Eur J Hum Genet. 2015;23(1):124–31. ejhg201450 [pii] 10.1038/ejhg.2014.50 . 24667786 PMC4266736

[74] Shi H, Qi X, Zhong H, Peng Y, Zhang X, Ma RZ, et al Genetic evidence of an East Asian origin and paleolithic northward migration of Y-chromosome haplogroup N. PLoS One. 2013;8(6):e66102 10.1371/journal.pone.0066102 PONE-D-13-08975 [pii]. . 23840409 PMC3688714

