# Supplementary material for: Revisiting the Diego Blood Group System in Amerindians: Evidence for Gene-Culture Comigration
Source: PLoS One. 2015 Jul 6;10(7):e0132211. doi: 10.1371/journal.pone.0132211 (PMC4493026; doi:10.1371/journal.pone.0132211)
Supplement: S1 File — (DOC) [file pone.0132211.s001.doc]

SUPPLEMENTARY INFORMATION

**Readme file for S1Table of Begat et al. " Revisiting the Diego blood group system in Amerindians: evidence for gene-culture comigration".**

S1Table presents the distribution of the 144 populations considered in the study according to the following cultural and environmental parameters:

- Latitude, longitude, N (population sample size), and *DI*A* allele frequency

Geographical coordinates, population sample size and allele frequency were compiled from the works listed in the "References" worksheet. When several works mentioned the same population, we simply estimated the weighted average of geographical coordinates and allele frequency, and summed the population sample sizes.

- Region

Broad geographic location was mentioned according to the following eight criteria: Subarctic, North America, Northwestern South America, Eastern South America, Andean, Lower Meso America, Chaco, and Central America.

- Ruhlen Linguistic classes

We used Ruhlen's linguistic classification [1,2] that divided the populations under study into 15 Native American language groups: Andean, Almosan, Chibchan, Equatorial, Eskimo-Aleut, Isolate, Keresiouan, Macro-Carib, Macro-Ge, Macro-Panoan, Na-Dene, Oto-Manguean, Paezan, Penutian, and Uto-Aztecan.

- Köppen-Geiger climate classes.

We labeled each population according the climate conditions they inhabit according to the updated Köppen-Geiger climate classification [4]: A-to-E: equatorial, arid, warm temperate, snow, polar; Am-Aw-Af: equatorial monsoonal, winter dry, fully humid; BS-Bs-Bw: arid steppe, summer dry, winter dry; Cs-Cw-Cf: warm temperate summer dry, winter dry, fully humid; Df: snow fully humid; ET: polar toundra.

- Main lifestyle

We assigned to each population the putative subsistence strategy they could have practiced during Pre-Columbian times: hunter-gatherer/forager, marine hunter-gatherer, or agriculturist [5-7].

- Regional maize species/cassava

We labeled each population depending on whether it inhabits an area with local crops of maize (*Zea* genus, core Andean South American, other South American, Guatemala and Southern Mexico, lowland western and northern Mexico, Eastern & Central USA, Southwestern USA, northern Mexico, ssp. *Parviglumis*, ssp. *Mexicana* [8]), and/or cassava (*Manihot* genus) [9], or not.

- Anopheline species

We labeled each population depending on whether it inhabits an area of one of combined species of mosquitoes as mapped in [10] or not. The eight anopheline species considered in the study are: 1- *Anophele quadrimaculatus*, 2-*A. pseudopunctipennis*, 3- *A. nuneztovari*, 4-*A. albimanus*, 5- *A. Albitarsis*, 6- *A. aquasalis*, 7- *A. darlingi*, 8- *A. marajoara*.

- Humanized landscape

Consideration was also given to variance among cultivated areas encompassing earthworks such as geoglyphs, raised fields, canals, causeways, large mounds and garden cities and regions inhabited non-earthmoving populations [11].

Lastly, population references used for computation of *DI*A* allele frequencies are listed in a separated worksheet entitled "References".

1. Greenberg JH, Ruhlen M (2012) An Amerind etymological dictionary; Department of Anthropology SU, editor. 312 p.

2. Ruhlen M (1987) A Guide to the World’s Languages, Vol. 1: Classification; Press SSU, editor.

3. Nordhoff SH, Harald & Forkel, Robert & Haspelmath, Martin (2013) Glottolog 2.2. Leipzig: Max Planck Institute for Evolutionary Anthropology Nordhoff, Sebastian & Hammarström, Harald & Forkel, Robert & Haspelmath, Martin (eds).

4. Kottek M, Grieser J, Beck C, Rudolf B, Rubel F (2006) World Map of the Köppen-Geiger climate classification updated. Meteorologische Zeitschrift 15: 259-263.

5. Gonzalez-Jose R, Garcia-Moro C, Dahinten S, Hernandez M (2002) Origin of Fueguian-Patagonians: an approach to population history and structure using R matrix and matrix permutation methods. Am J Hum Biol 14: 308-320.

6. Hunemeier T, Amorim CE, Azevedo S, Contini V, Acuna-Alonzo V, et al. (2012) Evolutionary responses to a constructed niche: ancient Mesoamericans as a model of gene-culture coevolution. PLoS One 7: e38862.

7. Steward JH, editor (1963) Handbook of South American Indians - Tome I, II, III, V, and VI. New York: Cooper Square. 818 p.

8. Matsuoka Y, Mitchell SE, Kresovich S, Goodman M, Doebley J (2002) Microsatellites in Zea - variability, patterns of mutations, and use for evolutionary studies. Theor Appl Genet 104: 436-450.

9. Leotard G, Duputie A, Kjellberg F, Douzery EJ, Debain C, et al. (2009) Phylogeography and the origin of cassava: new insights from the northern rim of the Amazonian basin. Mol Phylogenet Evol 53: 329-334.

10. Sinka ME, Rubio-Palis Y, Manguin S, Patil AP, Temperley WH, et al. (2010) The dominant Anopheles vectors of human malaria in the Americas: occurrence data, distribution maps and bionomic precis. Parasit Vectors 3: 72.

11. Mann CC (2005) 1491: New Revelations of the Americas Before Columbus; books Fv, editor. New York. 541 p.
