# Supplementary figures and images for: Revisiting the Diego Blood Group System in Amerindians: Evidence for Gene-Culture Comigration
Source: PLoS One. 2015 Jul 6;10(7):e0132211. doi: 10.1371/journal.pone.0132211 (PMC4493026; doi:10.1371/journal.pone.0132211)

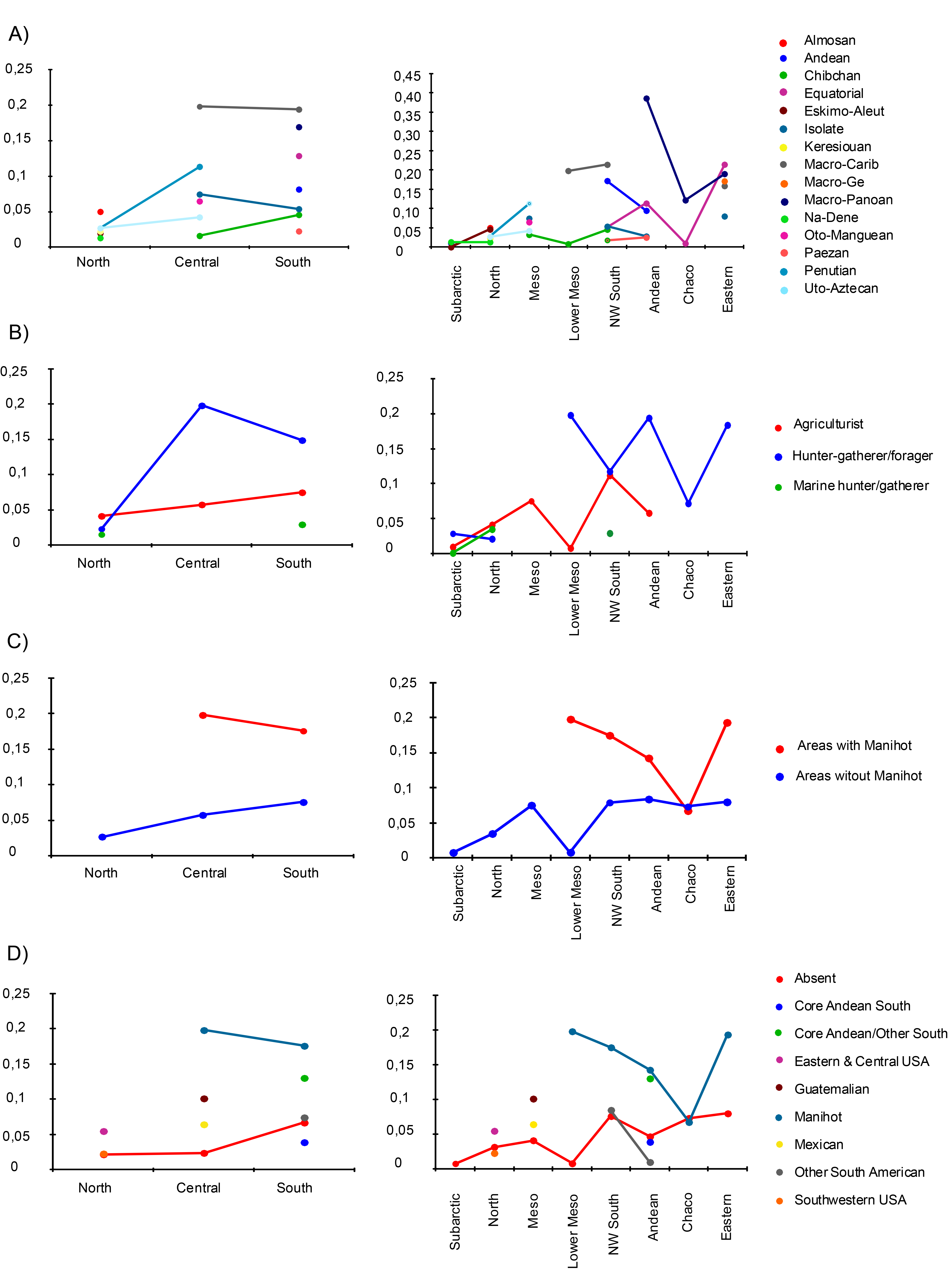

Supplement: S1 Fig — Left panels: three main areas where controlled. Right panels: eight main areas where controlled. Ruhlen's linguistic classes (Figure A). Main Pre-Colombian subsidence strategies (Figure B). Areas with/without cassava (Figure C). Areas with/without maize or cassava crops (Figure D). See S1 Table and S1 File for complete references. (TIF) [file pone.0132211.s003.tif]

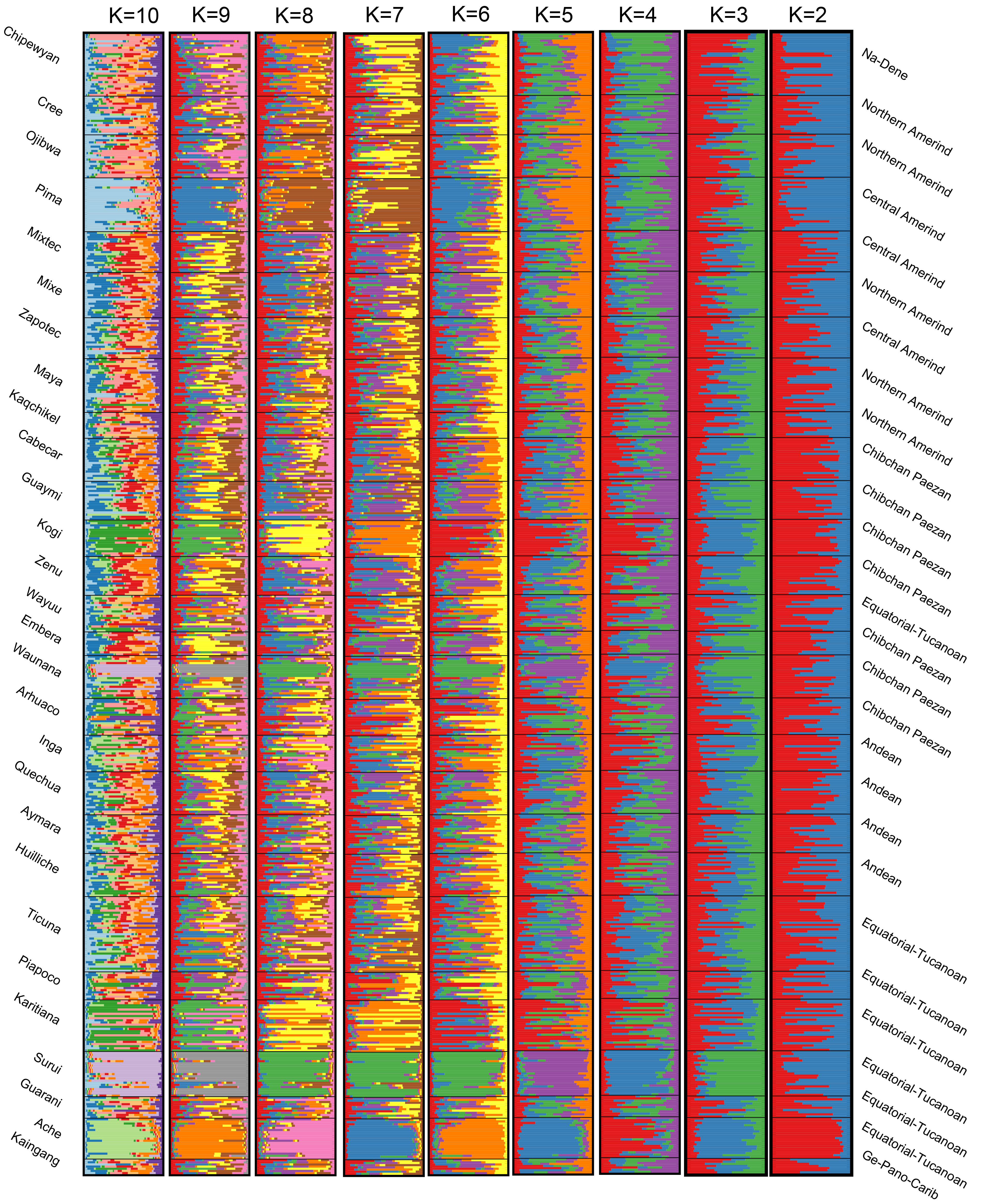

Supplement: S2 Fig — Samples were genotyped by [37]. (TIF) [file pone.0132211.s004.tif]
